# Ayahuasca-induced personal death experiences: prevalence, characteristics, and impact on attitudes toward death, life, and the environment

**DOI:** 10.3389/fpsyt.2023.1287961

**Published:** 2023-12-19

**Authors:** Jonathan David, José Carlos Bouso, Maja Kohek, Genís Ona, Nir Tadmor, Tal Arnon, Yair Dor-Ziderman, Aviva Berkovich-Ohana

**Affiliations:** ^1^Edmond J. Safra Brain Research Center, University of Haifa, Haifa, Israel; ^2^Integrated Brain and Behavior Research Center (IBBRC), University of Haifa, Haifa, Israel; ^3^Department of Counseling and Human Development, Faculty of Education, University of Haifa, Haifa, Israel; ^4^International Center for Ethnobotanical Education, Research & Service (ICEERS), Barcelona, Spain; ^5^Medical Anthropology Research Center (MARC), Department of Anthropology, Philosophy and Social Work, Universitat Rovira i Virgili, Tarragona, Spain; ^6^Department of Neurosciences and Behavior, University of São Paulo, São Paulo, Brazil; ^7^Integral Transpersonal Psychology, California Institute of Integral Studies, San Francisco, CA, United States; ^8^Department of Learning and Instructional Sciences, Faculty of Education, University of Haifa, Haifa, Israel

**Keywords:** ayahuasca, psychedelics, death, self, environmental concern, coping, life fulfillment

## Abstract

**Introduction:**

Despite an emerging understanding regarding the pivotal mechanistic role of subjective experiences that unfold during acute psychedelic states, very little has been done in the direction of better characterizing such experiences and determining their long-term impact. The present paper utilizes two cross-sectional studies for spotlighting – for the first time in the literature – the characteristics and outcomes of self-reported past experiences related to one’s subjective sense of death during ayahuasca ceremonies, termed here Ayahuasca-induced Personal Death (APD) experiences.

**Methods:**

Study 1 (*n* = 54) reports the prevalence, demographics, intensity, and impact of APDs on attitudes toward death, explores whether APDs are related with psychopathology, and reveals their impact on environmental concerns. Study 2 is a larger study (*n* = 306) aiming at generalizing the basic study 1 results regarding APD experience, and in addition, examining whether APDs is associated with self-reported coping strategies and values in life.

**Results:**

Our results indicate that APDs occur to more than half of those participating in ayahuasca ceremonies, typically manifest as strong and transformative experiences, and are associated with an increased sense of transcending death (study 1), as well as the certainty in the continuation of consciousness after death (study 2). No associations were found between having undergone APD experiences and participants’ demographics, personality type, and psychopathology. However, APDs were associated with increased self-reported environmental concern (study 1). These experiences also impact life in profound ways. APDs were found to be associated with increases in one’s self-reported ability to cope with distress-causing life problems and the sense of fulfillment in life (study 2).

**Discussion:**

The study’s findings highlight the prevalence, safety and potency of death experiences that occur during ayahuasca ceremonies, marking them as possible mechanisms for psychedelics’ long-term salutatory effects in non-clinical populations. Thus, the present results join other efforts of tracking and characterizing the profound subjective experiences that occur during acute psychedelic states.

## 1 Introduction

Psychedelic substances such as psilocybin, lysergic acid diethylamide (LSD), and ayahuasca (a potent Amazonian brew containing N,N-Dimethyltryptamine and Harmala alkaloids) have gained considerable popular and scientific attention in recent years (1). Their potential use as adjuncts for treating various mental health conditions has been recognized (2), with institutions such as the U.S. Food and Drug Administration (FDA), even granting them breakthrough therapy designations (3). Research on psychedelics has also centered around the profound experiences they induce, rendering them uniquely situated from advancing research on the phenomenology and neurophysiology of consciousness and the self (4). Common acute experiential effects of psychedelics include intense visual and auditory hallucinations, alterations in perception, changes in mood and thought processes, a shift in the sense of self, as well as a profound feeling of being transported to an alternative reality or dimension (5–7). Some of these experiences are so deeply profound and personally significant that they have been referred to using words such as ‘mystical’ (8) or ‘spiritual’ (9). Among these factors, the phenomenon of ‘ego dissolution’, marked by a profound sense of unity and the dissolution of boundaries between the self and the external world, has garnered significant attention (10).

The effects of these profound experiences outlast the acute state (11). The prevailing view among researchers is that such experiences are the primary drivers of psychedelics’ long-term effects [(12); but see (13, 14)]. This perspective is supported by a growing body of research, which has revealed a robust correlation between the quality of subjective experiences during psychedelic use and the enduring changes observed in human beliefs, emotions, and behavior over the long term (11). Multiple subjective effects, including but not limited to mystical-type experiences, ego dissolution, feelings of connectedness, psychological flexibility and emotional breakthroughs, have been found to be associated with the enduring changes observed over the long term (11, 15). For example, research has shown that the mystical-type acute experience which is characterized by a sense of transcendence and unity, along with ineffable and noetic qualities (16) is linked to increased long-term interpersonal closeness, a heightened sense of life meaning and purpose, shifts in coping strategies (17), increased belief in the transcendence of death (17–20), enhanced connection with nature (21), as well as a decrease in symptoms of depression and addiction behavior (22, 23). Such findings highlight the potential of psychedelic-induced experiences in bringing about profound changes in the individual’s relationship with the extended world on social, environmental, and spiritual levels – thus enhancing one’s sense of meaning in life and changing his relationship with it.

Another intriguing but underexplored profound experience that may arise during acute psychedelic states is the experience of ‘personal death’. In Ayahuasca-induced Personal Death (APD) experiences, an individual may feel an overwhelmingly authentic and convincing sensation of acutely dying or being dead, to the extent that it becomes indistinguishable from the ‘actual’ experience of dying or death (24, 25). APDs may be accompanied by anxiety and confusion (26) or/and with the experience of rebirth, salvation, mystical experience and the feeling of knowing what happens after death (25, 27). Reports of this phenomenon are well-documented in the psychedelics literature (5, 24–33), and it is widely believed that such experiences significantly impact users by allowing a deep realization of the human vulnerability and impermanence, potentially leading to radical personal transformations (24–26, 29). These transformations, as described by Grof and Halifax (24), frequently involve a profound realignment of core values. As a result, aspirations for worldly success, competitive drive, and the relentless pursuit of status, power, fame, prestige, and possessions often lose their allure. Simultaneously, these experiences often serve as gateways to the exploration of spiritual and religious dimensions, providing individuals with profound insights into the significance of the spiritual realm and its relevance within the broader context of existence.

While death experiences are not unique to any particular psychedelic substance, cultural, phenomenological, and pharmacological perspectives suggest they have a special affinity with ayahuasca. Culturally, the link between ayahuasca and the theme of death is present already in its original indigenous Amazonian context (25, 34), where the word ayahuasca means in Quechua *the dead-liana* (35) or *the vine of death* (36). Phenomenologically, some of the groundbreaking work regarding the phenomenology of ayahuasca was conducted by Benny Shannon based on a corpus of some 2,500 ayahuasca experiences descriptions summarized in the book “The Antipodes of the Mind” (25). Shannon recognizes the importance of APDs as some of the most potent and transformative experiences related to the brew and describes death-related experiences as some of the most prevalent cross-cultural subjective themes associated with the brew. Pharmacologically, the ayahuasca brew and its compounds, and in particular the N,N-Dimethyltryptamine (DMT), are related to experiences associated with the feeling of dying such as Near Death Experiences (NDEs) in terms of their phenomenology (7, 32, 37) and long term outcomes (25, 32, 38–40). Ayahuasca users, compared to other psychedelic users, report higher scores on questionnaires adapted from the NDE literature (32). The similarity between DMT/ayahuasca experiences and NDEs has even led to a popular theory that endogenous DMT is released during the dying process (31), a theory which has been met with skepticism by other researchers (41). For a recent review of the endogenous role of DMT in mammals see (42). However, it is important to note that other psychoactive drugs have also been proposed to elicit experiences that resemble NDEs (43, 44).

In sum, given the transformational and therapeutic efficacy of profound psychedelic acute experiences, and the cultural, phenomenological, and pharmacological affinity of ayahuasca to personal death experiences, investigating the APD phenomenon is timely. Thus, the primary objective of the current paper is to empirically explore, for the first time in the literature, the prevalence, characteristics, and long-term outcomes of APDs. It consists of two cross-sectional studies. Study 1 is a preliminary study of veteran ayahuasca users (*n* = 54) aiming at measuring the lifetime prevalence and characteristics of past APDs, as well as their association with death-related beliefs, attitudes, and connection with the extended world on social and environmental levels. Study 2 is a larger and more representative internet-based study (*n* = 306) aiming at generalizing Study 1’s findings regarding the prevalence and characteristics of APDs, and in addition, exploring the long-term associations of lifetime APDs with life engagement on the levels of both life values, as well as the ability to cope with difficult life situations. It is important to clarify that while both studies shared items allowing the basic characterization of APDs, their overall aims varied as each was part of a larger project associated with the neurophenomenology of death processing (Study 1) and public health (Study 2) in ayahuasca users in Israel. The study conforms to the Strengthening the Reporting of Observational Studies in Epidemiology (STROBE) reporting guideline for cross-sectional studies (45).

## 2 Study 1

Study 1 was designed as an initial, proof-of-concept exploration of the APD experience. It is part of a larger project studying death processing in ayahuasca veterans using phenomenology, neural and behavioral tasks, and self-report measures. Here, our aim was to provide the literature’s first characterization of lifetime APD experiences in terms of prevalence and intensity. As the prevalence of APDs is unknown, to increase the chances of detection we recruited participants who had significant experience with ayahuasca. To be able to link our results specifically to ayahuasca, only participants who considered ayahuasca as their main psychedelic were recruited. We also aimed at examine the effects of having undergone such experiences on attitudes toward death (46), and anxiety of death (47). We examined whether having such experiences was associated with psychopathology, and whether it could be predicted by certain personality traits – previously linked with other phenomenological aspects of the psychedelic experience (48) and NDE (49). Finally, we examined whether such past ‘*in vivo* experiences of dying’ (50) would soften one’s self-focus thus extending cognitive resources and emotional concern toward beyond-personal facets of life, including the social world (other people) and the natural world (environmental concern). The former was assessed using a well-established self-other bias perceptual task (51), and the latter via the gold standard in measuring environmental attitudes (52).

Based on the anecdotal evidence linking ayahuasca to the theme of death, we hypothesized that APD experiences (our independent variable) would regularly occur among veteran ayahuasca users, but could not make more precise hypotheses regarding actual rates due to the lack of previous structured inquiry. We also hypothesized that APD experiences would be retrospectively reported as being typically powerful and impactful and would be associated with stronger perceptions of having transcended death and reduced death anxiety (dependent variables). As psychedelic usage is closely linked with non-materialistic ontological beliefs (53), we did not expect categorization (continuation vs. annihilation) differences related to APD experiences due to a ceiling effect. We did, however, explore whether the degree of certainty in these views (as a dependent variable) were linked with past APDs. Possible associations between lifetime APD experiences and various exploratory control variables such as demographic factors, personality types, and psychopathology measures (depression, anxiety, and depersonalization) were examined. Finally, we hypothesized APD experiences to be associated with an increase in beyond-personal cognitive processing and concern (dependent variables), expressed as a reduction in the degree of behavioral bias toward ‘self’ (increased accuracy and reduced response times, see measures below) in comparison to ‘other’, as well as increased self-reported concern toward the environment. Importantly, to distinguish APD-specific effects from the ego dissolution literature, we also administered the Ego Dissolution Inventory (EDI) (54) as a dependent measure controlling of mystical experiences in general.

### 2.1 Materials and methods

#### 2.1.1 Participants

Fifty-four experienced ayahuasca users were recruited via social media and personal connections. Inclusion criteria were: multiple ayahuasca usage (>8 times), consideration of ayahuasca as their main psychedelic substance-of-use (their “medicine” of choice using the participants’ jargon), and no use of ayahuasca or other psychedelics in 28 days preceding the assessment, willingness to sign the informed consent, no current use of psychoactive medications (antidepressants, mood stabilizers, anxiolytics, and antipsychotics), no current use of drugs of abuse (cocaine), and less than twice a month of cannabis intake, no neurological or active psychiatric illnesses (e.g., epilepsy, depression), and no loss of a first degree relative (spouse, parent, child, or sibling) within the last 12 months. As the study was part of a larger neurophenomenological project, inclusion criteria included also Magnetoencephalography-compatibility factors including no history of head injury with loss of consciousness, not pregnant or lactating, and no claustrophobia or metal implants. Considerations relating to the larger project, neuroscientific ones in particular, also determined the study’s sample size. The study was approved by the Institutional Review Board of the Education Faculty, the University of Haifa, Israel. Together with other parts of the study not detailed here, participants spent around 4 h at home and in the lab completing the tasks and were compensated for their time by a sum of 100 Euros.

#### 2.1.2 Measures

##### 2.1.2.1 Demographics, personality, and ayahuasca use habits

*Demographic* items included age, gender, marital status, income, and education.

*Personality* was assessed using the Big Five Inventory [BFI, (55)], a commonly used personality assessment instrument that measures the five major personality traits, including openness, conscientiousness, extraversion, agreeableness, and neuroticism. The inventory consists of 44 items rated on a 5-point Likert scale (ranging from 1 – strongly disagree to 5 – strongly agree). Scores for each trait are calculated by averaging scores for the relevant items, with higher scores indicating higher levels of the trait. We used a well-established Hebrew version of the BFI (56). The resulting Cronbach’s alpha coefficients for BFI sub-scales extraversion, agreeableness, conscientiousness, neuroticism, and openness were 0.71, 0.71, 0.55, 0.83, 0.72, respectively.

*Ayahuasca use habits* included items on lifetime use, age of first ayahuasca consumption, time (in months) since last ayahuasca intake and since strongest ayahuasca experience. We also collected data regarding the lifetime usage of other psychedelics (Psilocybin, LSD, Mescaline).

##### 2.1.2.2 APD experiences: lifetime prevalence and characteristics

*APD experiences* were probed using 4 questions asking participants whether they (1) had personal death experiences during ayahuasca ceremonies (yes/no). If yes, participants were further asked (2) how many times they had such experiences (3-level ordinal scale of 1–5, 6–10, more than 10 times), (3) how intense these experiences were (Visual Analog Scale (VAS) ranging from 1 to 100, with 1 indicating “not intense at all” and 100 indicating “the most intense possible”), and (4) whether these experiences changed their attitudes toward death (1–4 point scale ranging from “no change” to “changed extremely). The term “intensity” was used as in prior research in the field (54, 57), with the goal of assessing the overall intensity of these experiences (which can differ across various senses, affectivity, valence, and spiritual tone).

##### 2.1.2.3 Ego dissolution

*Ego dissolution* was measured by the Ego Dissolution Inventory (EDI) (54). EDI is a 16-item self-report measure that assesses past ego dissolution experiences. Each item in the inventory is rated using a visual analog scale format (0–100, with incremental units of one) with zero defined as “No, not more than usually” and 100 defined as “Yes, entirely or completely. As in the original article, we used 2 EDI scales evaluating the strength of the: (1) “most intense” Ego Dissolution experience (EDI-S); (2) a “typical” Ego Dissolution experience (EDI-T). Total ego dissolution score was calculated by averaging the scores for all items, with higher scores indicating a greater level of ego dissolution. The EDI was translated as described in Section 2.1.3. The resulting Cronbach’s alpha was 0.93, matching the one reported in the original EDI English study (54).

##### 2.1.2.4 Death anxiety, beliefs, and attitudes

*Death anxiety* was gauged via the Death Anxiety Scale [DAS, (47)]. The DAS is the most widely-used and validated tool applied in both clinical and research settings for assessing death anxiety levels and its impact on individuals’ functioning. We employed the Likert scale version (58, 59) which consists of 15 items rated as a 1–5 Likert scale, ranging from 1 (strongly disagree) to 5 (strongly agree). The total score is calculated by averaging the scores for all items, with higher scores indicating a greater level of death anxiety. The DAS was translated as described in section 2.1.3. The resulting Cronbach’s alpha coefficient was 0.78, in the range of other studies using the translations of the scale to other languages (58).

*Ontological Afterlife Beliefs* (ALB) were probed via an in-house two-part self-report question indexing dis/belief in the continuation of soul/consciousness after death, as well as degree of certainty in the belief. Participants chose which of the following statements better suited their beliefs: “when the heart and brain stop working the soul/consciousness terminally ends” or “The soul/consciousness continues on after death.” Then, the participants rated their level of certainty in their answers using a VAS ranging from 0–100. A rating of 1 indicated “I do not know” while a rating of 100 indicated “I’m totally sure.”

*Death transcendence attitudes* were probed via the Death Transcendence Scale (DTS, Vandecreek, 1999). The DTS is a validated scale that measures death transcendence-attitudes and adaptations to the finitude of life and the sense of continuity after death. It includes 26 questions and five factors/subscales (mysticism, religion, nature, creativity, and biosocial). Importantly, the latter three subcategories are agnostic regarding the continuation of soul/consciousness as measured by the ALB scale. Recent research has shown increases in DTS scores following psychedelic Interventions (17–20). Each item is rated on a 5-point Likert scale (ranging from 1 – strongly disagree to 5 – strongly agree). The total score is calculated by summing the scores for all items, with higher scores indicating higher levels of death transcendence. The DTS was translated as described in section 2.1.3. The resulting Cronbach’s alpha coefficient was 0.69, in tide with the original published English version (Cronbach’s alpha = 0.74) (46).

##### 2.1.2.5 Psychopathology measures

*Depression* was measured via the Beck Depression Inventory [BDI, (60)]. The BDI is a validated tool commonly used in both clinical and research settings to assess the severity of depression and monitor changes in depressive symptoms over time. The measure consists of 21 items, with each item rated on a 0 to 3 scale, with higher scores indicating greater levels of depression (ranging from 0–63). Here we used a validated BDI Hebrew version (61). The resulting Cronbach’s alpha coefficient was 0.69, somewhat lower than described in literature (0.86) (62), but still within the range of reliability.

*Anxiety* was measured with the State–Trait Anxiety Inventory-Trait measure [STAI, (63)]. The STAI is a widely used and validated tool for assessing general anxiety level over time in both clinical and research settings. It consists of 20 self-report items, each rated as a 1–4 Likert scale, with higher scores indicating higher levels of anxiety. Scores are summed to obtain a total score (ranging from 20–80). We used a Hebrew version of the STAI questionnaire (64). The resulting Cronbach’s alpha coefficient was 0.91, well within the range of the original published scale (63).

*Depersonalization* was measured via the Cambridge Depersonalization Scale [CDS, (65)]. The CDS includes 29 self-report items rated on two Likert scales for frequency (1–4, ranging from never to all the time) and duration (0–10, ranging from a few seconds to more than a week) of experience. The total score on the CDS is calculated by summing the scores for all items in their respective subscales for frequency and duration, each of which yields a separate score. Higher scores on either subscale indicate greater degrees of depersonalization. The CDC was translated as described in Section 2.1.3. The resulting Cronbach’s alpha coefficient was 0.87, somewhat lower than reported in other translations of this scale but still highly reliable (66).

##### 2.1.2.6 Beyond-personal (others/nature) processing

*Relation to nature* was gauged via the New Ecological Paradigm Scale Revised [NEP-R, (67)] a self-report scale measuring environmental concern. The scale consists of 15 items rated on a 5-point Likert scale (ranging from 1 – strongly disagree to 5 – strongly agree). Total score is calculated by averaging the scores for all items, with higher scores indicating stronger pro-environmental attitudes. The NEP-R was translated as described in Section 2.1.3. The resulting Cronbach’s alpha was 0.78 in line with the original English version (Cronbach’s alpha = 0.81) (68).

*Relation to others* was assessed via the Self Prioritization Task [SPT, (51)]. The SPT task measures self-prioritization, which refers to the degree to which individuals exhibit an implicit behavioral bias toward the “self” as opposed to the “other.” Participants are trained in learning associations between geometric shapes (a triangle, a square, and a circle) and words ‘self’, best ‘friend, and an unfamiliar ‘stranger’. For example, they may be told “imagine your good friend is the circle, you are the triangle, and a stranger is the square.” Numerous studies (69) reliably show that when shapes are adapted to the “self” label, as opposed to being matched to the “familiar” or “stranger” labels, participants exhibit greater accuracy and faster response times, termed self-prioritization effects.

#### 2.1.3 Procedure

*Questionnaires* were completed online via a web link to an anonymous survey using the Qualtrics survey tool (Qualtrics, Provo, UT) between the 10.2021–06.2022. Questionnaires for which validated Hebrew translations were not found were translated in Hebrew using a back-translation process (70) for ensuring accurate and culturally appropriate meaning for Hebrew speakers. The process entailed one translator translating each questionnaire from English to Hebrew, and then another independent translator translating them back from Hebrew to English. A third independent translator, who was also a professional translator and editor, then evaluated the translations to identify and correct any discrepancies or errors.

*The SPT behavioral task* was administered at the Electromagnetic Brain Imaging Unit at Bar-Ilan University, Israel. The SPT task was presented using E-prime 3.0 on a 15.6-inch HD (1,366 × 768) screen on a Dell Vostro Laptop. The SPT task materials and procedure followed published guidelines (51), with the exception of being shortened to 240 trials administered in 4 60-trial rounds (compared to the original format of 3 rounds of 120 trials each). This was done following an in-lab pilot experiment which demonstrated no significant improvements in performance (reaction time and accuracy) beyond 240 trials.

After reading the task instructions on the computer screen and matching each shape to a label (counter-balanced across participants), the participants were given a short training session of twelve trials which were supervised by the experimenter to ensure participant task comprehension. Each trial started with a fixation cross appearing for 500 ms, after which a shape (triangle, circle, or square) and a word label (self, friend, stranger) were displayed for 100 ms above and below the cross, respectively. Immediately after that, a blank frame was presented for 1,300 ms in which time participants were required to judge whether the shape-label pair matched or not, by pressing one of the two response buttons as quickly and accurately as possible. Feedback (correct or incorrect) was immediately presented on the screen for 500 ms. At the end of each block, a frame was displayed informing the participants of their overall accuracy in the block. Responses longer than 1,300 ms were classified as misses and responses faster than 200 milliseconds were excluded from the analysis. Task outcomes were the participants’ d-prime (d’) accuracy scores and response times (RTs) of correct categorizations. Higher d’ and shorter RT scores indicate stronger self prioritization effects.

#### 2.1.4 Statistical analyses

Data analysis was conducted using SPSS version 23.0 (IBM Corp., Armonk, NY, USA) and jamovi software (version 2.3.1, The jamovi project). The internal consistency of the self-report scales was assessed using Cronbach’s alpha coefficient (71). Between-group (those who experienced or had not experienced APDs, named yAPD and nAPD, respectively) comparisons were performed using two-tailed independent *t*-tests for parametric data, Mann–Whitney *U* tests for non-parametric data, and chi-square tests for categorical data. Pearson’s (*r*) or Spearman’s (*ρ*) correlation coefficients were used (depending on ordinal/continuous nature of the data and its distribution) for exploring relations between APD features and other measures, as well as controlling for the more general mystical experience of ego dissolution. Normality was assessed via the Shapiro–Wilk test, and a significance level of less than 0.05 was used to determine departures from normality. The SPT task was assessed via mixed Anovas (for the d’ and RT dependent variables separately), with *identity* (self/friend/stranger) as a within-subject factor and *group* (yAPD/nAPD) as a between-subject factor, and *post hoc* analyses were conducted using *t*-tests. Effect size measures were calculated using Cohen’s d (d) and Eta squared (η2) for parametric data, and rank biserial correlation (r_p_) for non-parametric data. Significance values equal to or smaller than 0.05 were considered statistically significant.

### 2.2 Results

#### 2.2.1 Participants characteristics

Table 1 provides a summary of the study sample’s characteristics, including demographic variables, ayahuasca use parameters, personality traits, and psychopathology measures. The table also demonstrates a comparison between the yAPD and nAPD groups across these variables. The results show no statistically significant differences between the two groups in terms of ayahuasca use parameters, personality traits, and psychopathology measures. Supplementary Table S1 displays participants’ lifetime use of psychedelics (ayahuasca, LSD, Psilocybin, Mescaline) and includes a comparison between the groups in relation to lifetime use of other psychedelic. Results reveal a significantly higher use of ayahuasca compared to other psychedelics, with no significant differences between yAPD and nAPD groups for other psychedelics usge. Briefly, on average, our study participants have used ayahuasca (55.7 ± 82.1), 5.2 times more than psilocybin (mean = 10.7 ± 15.4, *U* = 1378, *p* < 0.01, *r*_p_ = 1), 4.6 times more than mescaline (mean = 12 ± 14.9, *U* = 351, *p* < 0.01, *r*_p_ = 1), and 5.6 times more than LSD (mean = 9.9 ± 16.6, *U* = 976, *p* < 0.01, *r*_p_ = 1).

**Table 1:** Summary of Study 1’s sample characteristics.

Variable	yAPD *n* = 36 (66.6%)		nAPD *n* = 18 (33.3%)	Total *n* = 54	Statistics (all n.s.)
**Demographics**					
Age		38.1 ± 7.5	39.4 ± 10.4	38.5 ± 8.6	*U* = 331
Gender	Male	25 (69.4%)	10 (55.5%)	35 (64.8%)	X^2^ = 1.02
	Female	11 (30.5%)	8 (44.4%)	19 (35.2%)	
Education	High School or equivalent	9 (25%)	1 (5.5%)	10 (18.5%)	X^2^ = 3.75
	College Diploma or certification studies	22 (61%)	12 (66.6%)	34 (63%)	
	Master’s Degree and above	5 (13%)	5 (27.7%)	10 (18.5%)	
Family status	Unmarried	18 (50%)	7 (38.8%)	25 (46.3%)	X^2^ = 6.25
	Married	12 (33%)	7 (38.8%)	19 (35.2%)	
	Divorced	6 (16.6%)	4 (22.2%)	10 (18.5%)	
Income	Below average	8 (22.2%)	4 (22.2%)	12 (22.2%)	X^2^ = 1.10
	Average	14 (38.8%)	9 (50%)	23 (42.3%)	
	Above average	14 (38.8%)	5 (27.7%)	19 (35.1%)	
**Ayahuasca parameters**	Lifetime use (number of times ayahuasca was taken)	69.4 ± 98.7	29.5 ± 13.7	55.8 ± 82.1	*U* = 255
	Age of first ayahuasca	30.4 ± 6.7	34.2 ± 10.5	31.7 ± 8.2	*T* = 107
	Last use (month)	6.7 ± 6.8	4.3 ± 5.2	8.2 ± 5.2	*U* = 252
	Most intense (month)	38.2 ± 41	24.9 ± 18.6	33.8 ± 35.5	*U* = 254
**Psychopathology**					
Depression (BDI)		5 ± 4.15	4.11 ± 4.17	4.7 ± 4.1	*U* = 279
Depersonalization (CDS)	Duration	45 ± 14.10	45.6 ± 12.04	45.2 ± 13.3	*U* = 566
	Frequency	46.1 ± 8.8	38.4 ± 9	45.6 ± 8.4	*T* = 150
Trait anxiety (STAI)		35.9 ± 8.6	38.4 ± 9	36.7 ± 8.7	*U* = 270
Personality (BFI)	Extraversion	3.4 ± 0.4	3.3 ± 0.6	3.44 ± 0.53	*T* = 0.741
	Neuroticism	2.42 ± 0.6	2.55 ± 0.6	2.44 ± 0.64	*T* = −0.668
	Agreeableness	3.93 ± 0.4	3.94 ± 0.3	3.94 ± 0.43	*T* = −0.146
	Conscientiousness	3.56 ± 0.4	3.50 ± 0.3	3.54 ± 0.40	*T* = 0.524
	Openness	4.12 ± 0.4	3.92 ± 0.5	4.04 ± 0.45	*T* = 1.53

The table summarizes the characteristics (means, SD and the statistics) of the study sample, including demographic variables, ayahuasca use parameters, personality traits, psychopathology, as a function of APD (yAPD group) or not (nAPD group). APD, Ayahuasca-Induced personal death; CDS, Cambridge personality scale; BDI, Beck Depression Inventory; BFI, Big Five Inventory; STAI, State–Trait Anxiety Inventory. Statistics: *T*, *T*-tests; *U*, Mann–Whitney tests; X^2^, chi-square tests. No significant between-group differences were found.

#### 2.2.2 APD experiences prevalence and characteristics

**Figure 1:**
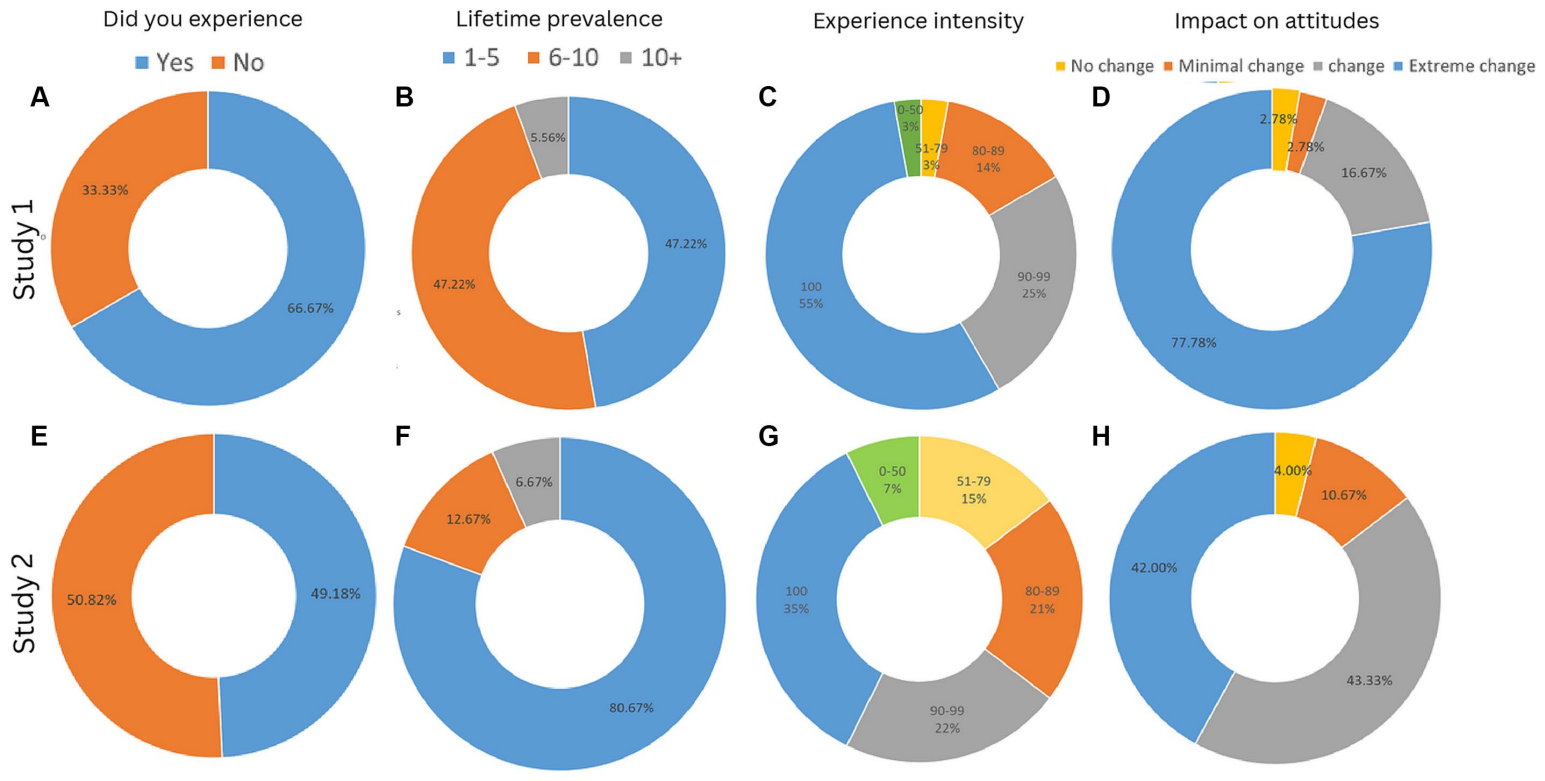
Prevalence and characteristics of APD experiences across Studies 1 and 2: Top row **(A–D)** displays results from Study 1 (*n* = 54), and bottom row **(E–H)** from Study 2 (*n* = 306). The overall occurrence (yes/no) of APD experiences are displayed in **(A,E)**. For those who had experienced APDs (*n* = 36 in Study 1, and *n* = 155 in Study 2), **(B,F)** displays their lifetime frequency rates, **(C,G)** displays their perceived intensity, and **(D,H)** their perceived impact on attitudes related to death.

Of the 54 ayahuasca users, 36 participants (66.7%) reported having experienced APDs while 18 participants reported not having experienced APDs (Figure 1A). Within the yAPD group, 17 participants (47.2%) reported experiencing it 1–5 times, 17 participants (47.2%) reported experiencing it 6–10 times, and 2 participants (5.6%) reported experiencing it more than 10 times (Figure 1B). In terms of the perceived intensity of the APD exeriences, the mean subjective intensity of APD experiences was 93.6 (SD = 10), and the median was 100, with only 2 participants reporting APD intensity below 80 (Figure 1C). In terms of subsequent change in attitude toward death, 28 participants (77.8%) reported an extreme change, 6 participants (16.7%) reported a moderate change, only one participant (2.8%) reported a minimal change, and another participant (2.8%) reported no change (Figure 1D).

#### 2.2.3 Death related anxiety, beliefs, and attitudes

**Figure 2:**
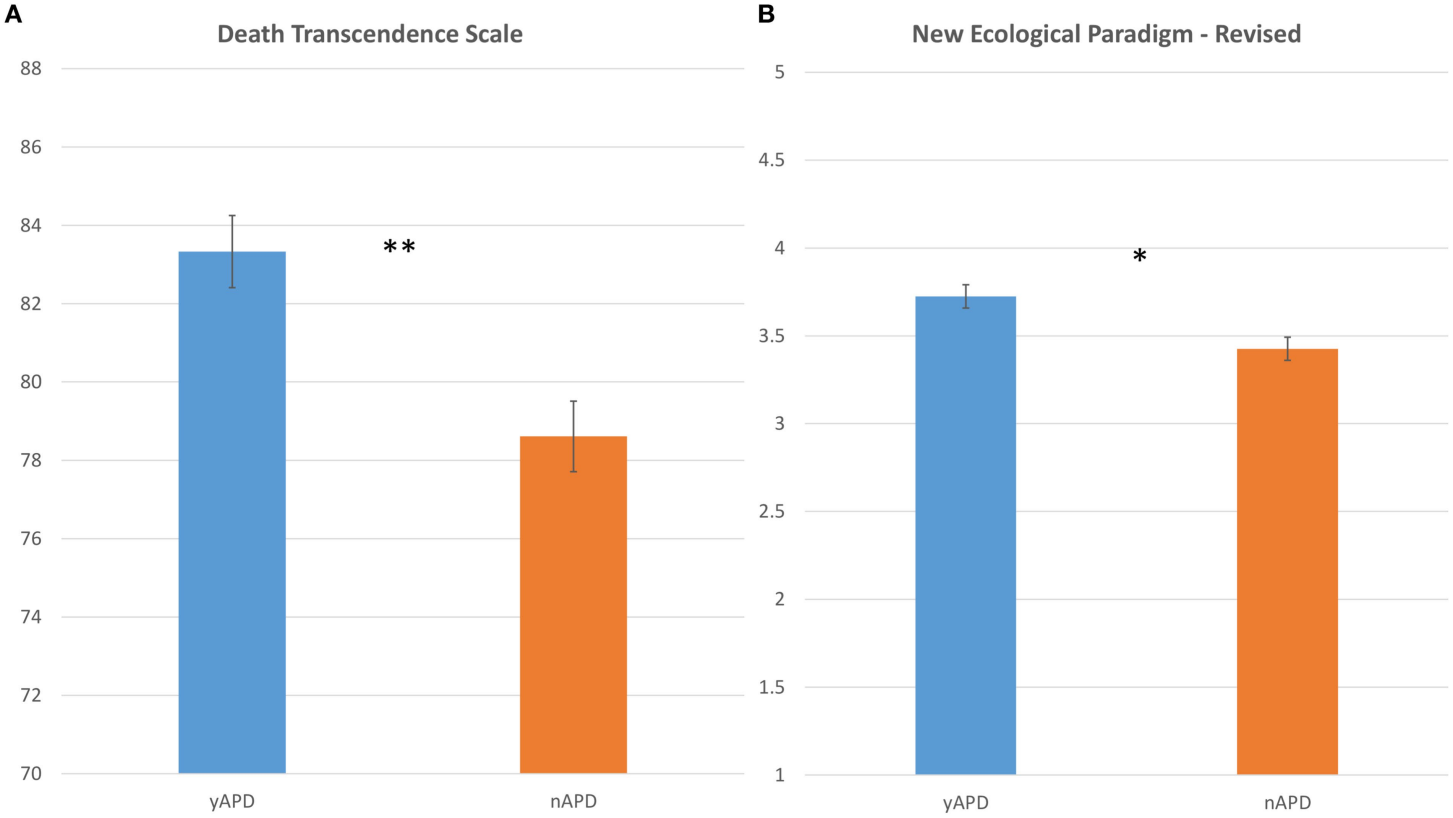
Death transcendence attitudes and environmental concern as a function of experiencing APDs. Bar plots comparing the distribution of **(A)** DTS scores (*y*-axis), and **(B)** NEP-R scores (*y*-axis), as a function of the yAPD group (in blue) and nAPD group (in orange). Error bars represent the standard error of the mean. DTS, Death Transcendence Scale, NEP-R, New Environmental Paradigm Revised. Statistics: *p*-values ≤ 0.01 are denoted by **, and *p*-values ≤ 0.05 are denoted by *.

In distinction to our hypothesis, no differences in death-related anxiety (DAS scores) were found between the yAPD (mean = 2.67 ± 2.67) and nAPD (mean = 2.67 ± 2.53) groups (*t*(52) = −0.01, n.s.). Regarding ontological afterlife beliefs, nearly all the participants (94.4%) endorsed a belief in the continuation of the soul/consciousness after death, with a high degree of certainty (mean = 86.6% ± 19, median = 90). Both measures did not differ between the groups. Regarding death-related attitudes, the findings demonstrated (Figure 2A) a significantly stronger endorsement (higher DTS scores) of death transcendence views [*t*(52) = 2.62, *p* = 0.01, *d* = 0.75] for the yAPD group (mean = 83.3 ± 6.2) relative to the nAPD (mean = 78.6 ± 6.2) group. In addition, within the yAPD group, the perceived impact of APD experiences on attitudes related to death strongly predicted the DTS scores (*ρ* = 0.575 *p* < 0.001). Finally, our analysis revealed also a significant correlation between the DTS score and the EDI-S scale (*ρ* = 0.410, *p* < 0.01) but not the EDI-T scale (*ρ* = 0.225, *p* < 0.102), thus suggesting our results may not be APD-specific.

#### 2.2.4 Beyond-personal (others/nature) processing

The results regarding the effect of APDs on beyond personal factors were mixed, indicating significantly higher [*t*(52) = 2.268, *p* = 0.02, *d* = 0.655] environmental concern (higher NEP-R scores) for the yAPD group (mean = 3.73 ± 0.46) relative to the nAPD group (mean = 3.43 ± 0.45) (Figure 2B), thus confirming our hypothesis regarding the link between APDs and environmental attitudes. Importantly, these results were specific to APD experiences. Both typical and strongest measures of ego dissolution did not predict environmental concern (EDI-S *ρ* = 0.218, *p* = n.s; EDI-T *ρ* = 0.087, *p* = n.s).

However, no significant differences were found for beyond personal social processing (d’ and RT in the SPT task). The SPT analysis included data from 51 participants (2 did not do the task, and one participant’s data was missing). A repeated-measures 2×3 ANOVA with *group* (yAPD/nAPD) and *identity* (self/friend/stranger) as within-participant factors was conducted for d’ and RT dependent variables. The results revealed highly significant main effects of *identity* for both accuracy [*F*(2,98) = 10.92, *p* < 0.001, η^2^ = 0.05] and RTs [*F*(2,98) = 28.31, *p* < 0.001, η^2^ = 0.145], with posthoc tests replicating the task’s self-prioritization effect. Participants displayed significantly higher d’ scores and faster RTs when responding to self-related stimuli compared to friend-related stimuli (d’, *p* < 0.001; RT, *p* = 0.006), compared to stranger-related stimuli (d’, *p* < 0.001; RT, *p* < 0.001). Contrary to our hypothesis, the *group* x *identity* interaction was not significant for both d’ [*F*(2,98) = 0.142, *p* = 0.886] and RT [*F*(2,98) = 0.536, *p* = 0.587], suggesting that self-prioritization was not affected by having experienced APDs.

## 3 Study 2

Study 2 had two aims. The first is generalize Study 1’s results regarding the prevalence and characteristics of lifetime APD experiences, while addressing its potential selection bias due to its small and unique sample of veteran ayahuasca users with extensive experience with the brew. Thus a larger, more representative internet-based sample of ayahuasca users with any degree of experience were recruited. The second aim was extending Study 1’s results by exploring the association between APD’s and life engagement. Study 2 aimed at exploring the association between having experienced APDs (independent variable) on the manner in which one engaged with life. Specifically, we focused on assessing the participants’ self-reported ability to cope with distressing situations, as well as their values in life (dependent variables). We hypothesized that similar to findings from NDE studies (38, 39, 72–75), APDs would be associated with an enhanced ability to cope with distressing life situations and increased life values and meaning.

### 3.1 Methods

#### 3.1.1 Participants

Participants were recruited as part of a larger study assessing the public health of ayahuasca users in Israel. 306 ayahuasca users were recruited via ayahuasca contact groups and psychedelic social media groups in Israel. Sample size determination was licensed by the medium to large between-subjects effect sizes reported in Study 1 (*d*s of 0.65 and 0.75 for the DTS and NEPR scales, respectively), as well as the discovered 2 to 1 ratio of participants who had not-experienced vs. experienced APDs. Thus, conservatively assuming a medium effect size of *d* = 0.5, and a 2 to 1 APD experience ratio, our sample size is sufficient for determining group differences (*n* = 236 allows power = 0.95 at α = 0.05 for *d* = 0.5, and a 2/1 allocation ratio). Inclusion criteria were: age > 18, at least six months since initial ayahuasca intake, be able to fluently read and understand Hebrew, and provided informed consent. The study protocol was approved by the Institutional Review Board of the Faculty of Education, University of Haifa, Israel. All participants provided written informed consent to participate in the study and did not receive financial compensation.

#### 3.1.2 Procedure

Data were collected via the Qualtrics online survey tool (Qualtrics, Provo, UT) using a web link to an anonymous survey between the 12.2022–02.2023. The back-translation procedure (70) was used for translating questionnaires not available in Hebrew. Participants dedicated around 30 min to complete an online questionnaire including demographics, ayahuasca consumption patterns, information on APD experiences (as in Study 1), and a comprehensive public health survey (to be reported elsewhere). In addition, each participant completed two questionnaires indexing coping style with distress and values in life [see (76, 77)].

#### 3.1.3 Measures

##### 3.1.3.1 Demographics and ayahuasca usage habits

*Demographic* information included age, gender, marital status, income, education, and lifetime psychedelics use (Psilocybin, LSD, mescaline).

*Ayahuasca use habits* included lifetime consumption amount, age of first ayahuasca intake consumption, time since last ayahuasca experience, and ayahuasca consumption settings.

##### 3.1.3.2 APD lifetime prevalence, characteristics, and ontological beliefs

*APD experiences* were probed using the same four questions as in Study 1, described previously.

*Ontological afterlife beliefs* were gauged using the ALB measure described previously.

##### 3.1.3.3 Life engagement and coping

*Coping strategies* were assessed using the Hebrew version, shortened (78) Coping Strategies Scale (79). The shortened COPE is a 30-item tool designed to evaluate two categories of coping strategies: problem-focused coping (COPE-p) and emotion-focused coping (COPE-e). Problem-focused coping strategies involve taking actions to manage or solve the distress causing problems (i.e., gathering information, making a plan, or seeking advice from others). Emotional-focused coping strategies involve managing the emotional distress caused by a stressful situation (i.e., seeking emotional support, distracting oneself from the stressor, or engaging in activities that help regulate emotions). Participants were asked to indicate how often they use each coping strategy when faced with a stressful situation, with items rated on a 4-point Likert scale, ranging from 0 (not at all) to 3 (very much). Higher scores indicate a greater use of the respective coping strategy. The resulting Cronbach’s alpha coefficient was 0.72 for COPE-p and 0.61 for COPE-e, close to the coefficient values published in the original COPE article (COPE-*p* = 0.76 and COPE-e = 0.64) (78).

*Values in life* were assessed via the Engaged Living Scale [ELS, (80)]. The ELS is a self-report scale measuring personal values and more specifically “engaged living,” as understood in the Acceptance and Commitment Therapy (ACT) model. The scale is composed of two subscales: valued living (ELS-v) and life fulfillment (ELSl-f). The ELS-v consists of 10 items assessing an individual’s ability to recognize and comprehend their values, as well as their capacity to engage in actions that are consistent with these values. The ELS-f includes 6 items that evaluate a person’s sense of fulfillment in life as a result of recognizing and living in accordance with their values. ELS items are rated on a 5-point Likert scale, ranging from 1 (completely disagree) to 5 (completely agree), with higher scores indicating greater levels of engaged living. The ELS was translated as described in section 2.1.3. The resulting Cronbach’s alpha coefficients were 0.89 for ELS-l and 0.86 for ELS-v, in line the coefficients reported in the original article (ELS-l and ELS-v = 0.86) (80).

#### 3.1.4 Statistical analyses

Data analysis was conducted using SPSS version 23.0 (IBM Corp., Armonk, NY, USA) and jamovi software (version 2.3.1, The jamovi project). Internal consistency of the self-report questionnaires was assessed using Cronbach’s alpha coefficient (71). Between-group comparisons (yAPD and nAPD participants) were performed using two-tailed independent-samples *t*-tests for parametric data, Mann–Whitney *U* tests for non-parametric data, and chi-square tests for categorical data. Effect sizes were calculated to provide additional information about the magnitude of the differences found. Cohen’s d (d) was used for parametric data and rank biserial correlation (*r*_p_) was used for non-parametric data. Pearson’s (*r*) or Spearman’s (*ρ*) correlation coefficients were used (depending on ordinal/continuous the nature of the data and whether it was normally distributed) to assess relations between the APD features and other hypothesized measures, as well as to rule out the effects of other ayahuasca parameters. Normality was assessed via the Shapiro–Wilk test, and a significance level of less than 0.05 was used to determine departures from normality. Significance values equal to or smaller than 0.05 were considered statistically significant.

### 3.2 Results

#### 3.2.1 Participants characteristics

Table 2 provides a summary of the study sample’s characteristics, including demographic variables and ayahuasca use parameters. The table also demonstrates a comparison between the yAPD and nAPD groups across these variables. One participant was excluded due to unreliable results (participant answered ‘1’ for all questions). In line with the results of Study 1, no significant group differences were observed in demographics. However, in contrast to Study 1, our findings revealed significant differences between the groups in terms of lifetime ayahuasca use, last ayahuasca intake, and age of first use with the brew. yAPD participants had been in more ayahuasca ceremonies, their last ayahuasca intake was more recent, and they had consumed ayahuasca for the first time earlier than the nAPD group. There was a slight difference between the yAPD and nAPD groups in usage parameters of other psychedelic substances (LSD and Mescaline, see Supplementary Table S2). However, we ensured via correlation analyses tests that these were not associated with the dependent variables. It is important to highlight that the participants in Study 2 were considerably less experienced users compared to those in Study 1. In Study 2, approximately 50% of the sample reported using ayahuasca between 1 and 10 times. On the other hand, in Study 1, the average intake was 69.4 and only one subject reported having consumed ayahuasca fewer than 10 times.

**Table 2:** Summary of the Study 2’s sample characteristics.

Variable		yAPD *n* = 150 (49.2%)	nAPD *n* = 155 (51.8%)	Total *n* = 305	Statistics
**Demographics**					
Age		41.4 ± 8.41	43.9 ± 11.4	43 ± 10.3	*U* = 10,494, n.s.
Gender	Male	69 (46%)	74 (47.7%)	143 (46.9%)	X^2=^0.433, n.s.
	Female	80 (53.3%)	79 (50.9%)	159 (52.1%)	
	Other^a^	1 (0.6%)	2 (1.2%)	3 (0.9%)	
Education	Primary school or less^b^		1 (0.6%)	1 (0.3%)	X^2=^11.7, n.s.
	High School or equivalent	69 (46%)	62 (40%)	131 (42.9%)	
	B.A	40 (26.6%)	51 (32.9%)	91 (29.8%)	
	Master’s Degree and above	39 (26%)	39 (25.1%)	78 (25.5%)	
Family status	Single	45 (30%)	36 (23.2%)	171 (56%)	X^2=^23.4, n.s.
	Married	81 (54%)	90 (58%)	40 (13.1%)	
	Divorced	16 (10.6%)	24 (15.4%)	81 (26.5%)	
	Other	8 (5.3%)	5 (3.2%)	13 (0. 4%)	
Income	Below average	54 (36%)	56 (36.1%)	110 (36%)	X^2=^34.2, n.s.
	Average	37 (24.6%)	32 (20.6%)	69 (22.6%)	
	Above average	50 (33.3%)	57 (36.7%)	107 (35%)	
**Ayahuasca parameters**					
	Local/neo-shamanic	55 (36.6%)	50 (32.2%)	105 (34.4%)	X^2=^4.29, n.s.
Setting	Religious, indigenous, or other	95 (63.3%)	105 (67.7)	200 (65.6%)	
Lifetime intake	1–10	56 (37.3%)	94 (60.6%)	150 (49.2%)	***U* = 8,539, *p* < 0.01**
	11–20	22 (14.6%)	22 (14.1%)	44 (14.4%)	
	21–50	29 (19.3%)	16 (10.3%)	44 (14.4%)	
	51–100	20 (13.3%)	13 (8.3%)	33 (10.8%)	
	100+	23 (15.3%)	11 (7%)	34 (11.1%)	
Last intake	Last month or less	48 (23%)	20 (12.9%)	68 (22.3%)	***U* = 9,280, *p* < 0.01**
	Past 1–3 months	25 (16.6%)	34 (21.9%)	59 (19.3%)	
	Past 3–6 months	21 (14%)	23 (14.8)	44 (14.4%)	
	Past 6 months to 1 year	18 (12%)	27 (17.4%)	45 (14.8%)	
	Past 1 year to 3 years	21 (26%)	27 (17.4%)	48 (15.7%)	
	More than 3 years ago	17 (11.3%)	14 (0.9%)	41 (13.5%)	
Age of first intake		33.8 ± 8.7	37.1 ± 11	35.5 ± 10	***U* = 9,530, *p* < 0.05**

The table presents data on demographic characteristics and ayahuasca use parameters for both the yAPD and nAPD groups. Bold markings indicate statistically significant findings. ^a^Only male and female participants were compared, as other gender identities were not included in the statistical analysis due to insufficient sample sizes for meaningful comparison. Likewise, ^b^The category “Primary school or less” was not included in the statistical analysis. ^*^Percentages reported do not always add up to 100% as some participants did not answer certain questions.

#### 3.2.2 APDs lifetime prevalence, intensity, and impact

Of the 305 ayahuasca users surveyed, 150 (49.2%) reported having experienced past APDs (Figure 1E). The large majority of the ayahuasca users (121 participants or 80.7%) experienced it between one and five times, whereas 19 participants or 12.7% experienced it between six and ten times, and only ten participants or 6.7% experienced it more than ten times (Figure 1F). The participants reported that the perceived intensity of the APD experiences was strong with a mean of 85.01 (SD = 20.3) and a median of 91 (Figure 1G). The APD was also perceived as impactful and changed their attitudes toward death. 63 participants (42%) reported an extreme change in their attitudes toward death, 65 participants (43.3%) reported a moderate change, 16 participants (10.6%) reported minimal change, and six participants (4%) reported no change in their attitudes toward death (Figure 1H). It should be noted that, in comparison to Study 1, the prevalence of APD was smaller and their intensity were less pronounced and impactful. These differences are likely attributable to the distinct sample characteristics of Study 1, where participants were more experienced with ayahuasca. However, we can not rule out that Study 1’s veteran sample may differ in other characteristics and psychological traits.

#### 3.2.3 Ontological afterlife beliefs

The ALB results indicated a clear bias toward literal immortality, as 92.5% of the participants endorsed the view that the soul/consciousness continued on after death. Participants were also very certain about their views (mean = 84.1 ± 23, median = 90). These results align with those of the sample in Study 1. In terms of group differences, there was no significant difference in ALB categorization between the yAPD and nAPD groups [X^2^ (1, *N* = 305) = 0.001, n.s.]. However, ALB certainty was higher (*U* = 9,472, *p* < 0.001, *r*_p_ = 0.185) in the yAPD group (mean = 87.8 ± 19.8) than nAPD group (mean = 80.5 ± 25.4).

#### 3.2.4 Life engagement and coping

**Figure 3:**
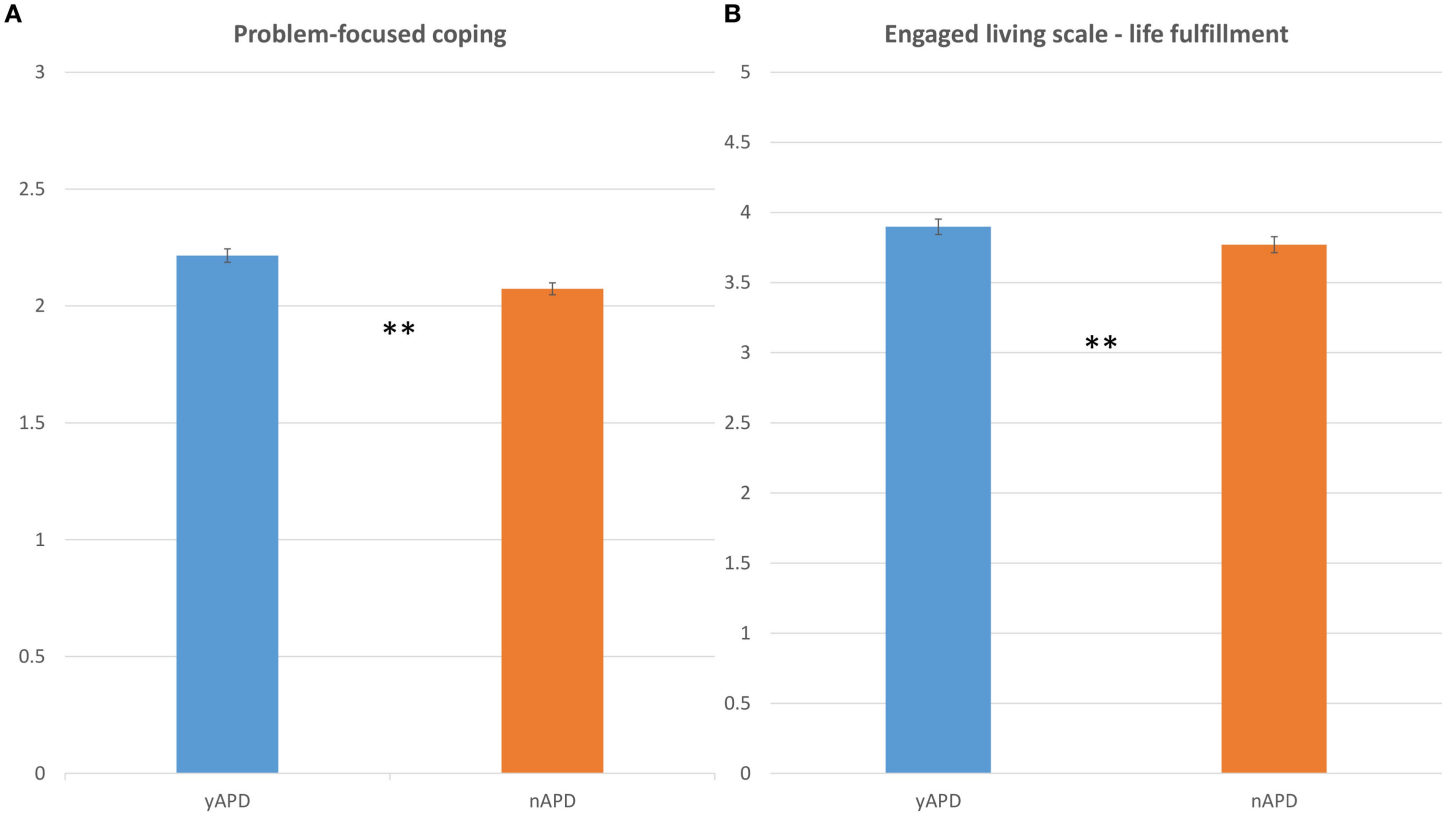
Life engagement and coping strategies as a function of APD. Bar plots comparing the distribution of **(A)** COPE-p scores (*y*-axis), and **(B)** ELS-f scores (*y*-axis), as a function of the yAPD group (in blue) and nAPD group (in orange). Error bars represent the standard error of the mean. COPE-p, Problem-focused coping; ELS-f, Engaged Living Scale-life fulfillment. Statistics: *p*-values ≤ 0.01 are denoted by **, and *p*-values ≤ 0.05 are denoted by *.

In terms of coping strategies, results were mixed. As predicted, yAPD participants demonstrated significantly higher scores (*U* = 8,823, *p* < 0.01, *r*_p_ = 0.24) on the problem-focused coping strategies with higher COPE-p scores (mean = 2.22 ± 0.31) compared to the nAPD group (mean = 2.07 ± 0.36) (Figure 3A). However, there was no significant group differences [*t*(303) = 1.08, *p* = 0.277, n.s.] in terms of emotional-focused coping strategies (COPE-e scores). Importantly, to ensure that these group differences could not be accounted for by sample differences in lifetime use rates, age of initial intake, and last intake, we ran correlation analyses between the COPE subscales and these variables. The results indicated no significant correlation (all *p*s > 0.1) between coping strategies and lifetime ayahuasca use (COPE-p, *ρ* = −0.02, n.s.; COPE-e, *ρ* = 0.0006, n.s.), initial intake age (COPE-p *r* = −0.005, n.s.; COPE-e *r* = −0.01, n.s.), and last intake (COPE-p *ρ* = −0.06, n.s.; COPE-e *ρ* = −0.004, n.s.). These findings align with previous results of some of the current authors, where no associations were found between the frequency of ayahuasca use and coping measures (76).

In terms of engaged living, results were mixed as well. As shown in Figure 3B, the yAPD group felt more fulfillment in life with significantly higher scores (*U* = 10,125, *p* = 0.05, *r*_p_ = 0.12) on the ELS-f subscale (mean = 3.9 ± 0.70) compared to the nAPD (mean = 3.7 ± 0.68). However, there were no significant differences (*U* = 10,417, *p* = 0.116, n.s.) observed between the groups in terms of life values (ELS-v scores). An additional correlation analysis within the yAPD group showed that the degree to which death-related attitudes were impacted by APD experiences predicted ELS scores (ELS-v, *ρ* = 0.365, *p* < 0.001; ELS-f, *ρ* = 0.231, *p* = 0.004), thus providing evidence that degree of engaged living was impacted by APD experiences. Again we controlled for group differences related to ayahuasca use parameters. No significant correlations (*p*s > 0.1) were found between the ELS subcales and lifetime ayahuasca intake (ELS-v: *ρ* = 0.08, n.s.; ELS-f: *ρ* = 0.01, n.s.), and only marginally significant correlations emerged between the ELS subscales and the age of initial ayahuasca intake (ELS-v: *r* = 0.10, *p* = 0.07; ELS-f: *r* = 0.13, *p* = 0.05). These results partially corroborate previous findings (76), which demonstrated a significant relationship between ELS-v (but not ELS-f) and lifetime ayahuasca use. A significant correlation was found between ELS-v and the last ayahuasca intake intake (ELS-v: *ρ* = −0.114, *p* = 0.04). Furthermore, marginally significant correlations emerged between ELS-f and last ayahuasca intake (*ρ* = −0.11, *p* = 0.052).

## 4 Discussion

The present study aimed at spotlighting, for the first time in the literature, death experiences occurring during ayahuasca ceremonies. In two independent studies, we examined their prevalence rates, experiential characteristics, and associations with death perceptions. Additionally, we examined the link between lifetime APDs and how the extended world was approached (Study 1), as well as on life values and coping strategies (Study 2).

Our findings indicate that APDs are a common experience among those participating in ayahuasca ceremonies, being reported by at least half of the participants. Having such experiences was not related to gender, age, education, personality, or ontological belief. However, while prevalent, these experiences were not very frequent with participants mostly experiencing them no more than 5 times over their lifetime, and very rarely more than 10 times. As expected, these experiences are perceived as powerful and impacted people’s attitudes toward death. In both studies, most participants rated APD experiences at the maximum intensity afforded by the scale, and most participants reported APDs to have significantly changed their attitudes toward death. These reports were further validated by other measures showing that lifetime APDs predicted having a stronger sense of having transcended death (in Study 1), and more certainty in the continuation of the soul/consciousness after death (in Study 2). However, in contrast to our expectations APDs did not influence death anxiety levels, and neither were they predictive of psychopathology including depression, anxiety, and depersonalization. In fact, as expected, participants who experienced APDs displayed better problem-solving life coping skills and perceived life as more fulfilling (Study 2). Finally, while APD experiences were not associated with less bias toward the self, in contrast to our expectations, they were associated with increased pro-environmental perceptions as expected (Study 1). Thus, these results establish APDs as frequent, profound, and transformative experiences which have the potency to impact the perception of – or relation to – life, death, and the environment. Important to note, there were differences between Study 1 and Study 2 concerning lifetime experience of APD, intensity, and impact—all of which are lower in Study 2. These variations can be attributed to the distinct sample characteristics of Study 1, where participants were more experienced and considered ayahuasca as their primary psychedelic medicine. Therefore, we postulate that the more one uses ayahuasca, the more possible a strong and transformative APD will be.

### 4.1 APDs and the perception of death

A structured phenomenological study of the APD experience is still lacking, however, certain anecdotal features gathered from the literature point at an extremely powerful and convincing experience. Participants describe such experiences as consisting of authentic and convincing feelings of dying or being dead, with them often losing the awareness of being in a psychedelic session and undergoing a symbolic experience (24, 25). Other experiential features which may accompany APDs include disembodiment aspects such as seeing oneself from above, the experience of rebirth, salvation, mystical experience, anxiety, confusion and the feeling of knowing what happens after death, while maintaining some self-awareness (25–27).

While APDs do not involve a real situation in which the experiencer is close to actual death, it is experienced that way, and there is evidence that there are similarities between ayahuasca and DMT and NDEs in terms of the phenomenology (5, 7, 31, 32). Similar to NDEs, the experiential realization that consciousness and awareness persist despite the sense of physical bodily death, the encountering mystical beings and other NDE elements may reinforce the belief that consciousness can exist independently of a living body, and even after death (81, 82). Hence, this realization may strengthen the conviction in the existence of an afterlife and may foster a deeper sense of transcendence in relation to death – in line with the results of the present study. Prior studies show a positive correlation between afterlife beliefs and psychological well-being (83–85), suggesting that these beliefs can liberate individuals from fundamental fears, avoidance patterns, and the continual need for self-worth validation (86–88). However, the impact of afterlife beliefs conduct depends on specific sets of beliefs (85, 89), and therefore, further studies are necessary for examining the specific manifestation of afterlife beliefs in ayahuasca users and their alteration following APD experiences.

While no links were found between APDs and psychopathology, and on the other hand, positive effects in terms of life coping and fulfillment were found, it is premature to classify APDs as inherently positive phenomena. Again drawing parallels from the body of literature concerning NDEs [(90), but see (91)] as well as anecdotal evidence related to psychedelics (92), reports indicate that a certain percentage of individuals undergoing profound experiences develop post-traumatic stress disorder symptomatology, alongside elevated levels of depression and anxiety. Several factors contribute to this outcome, including the possibility that some individuals fail to comprehend or contextualize the essence of these experiences within their existing worldviews. Consequently, they might experience a sense of losing touch with reality, accompanied by apprehension about sharing their experiences with friends and family members.

Previous studies have found analogous results with other psychedelics such as LSD and Psilocybin. Clinical trials involving the administration of these psychedelics have demonstrated an increase in DTS scores subsequent to the experiences, and these increases have been found to correlate with the intensity of acute mystical-type subjective effects (17–20). As our results also indicated a strong correlation between death transcendence and (strongest but not typical) ego-dissolution experiences, it may be the case that attitudes toward death are impacted more generally by strong mystical experiences and are not APD-specific. In addition, contrary to our predictions, death anxiety levels did not differ between those who experienced APDs or not, and were also not correlated with ego-dissolution. Thus, it is possible that there is a floor effect where a few experiences are sufficient for lessening death anxiety. This aligns with studies that illustrate a reduction in death anxiety following the use of psychedelics (32, 93). An alternative explanation is that some of the APD experiences may have been difficult and challenging. Thus, participants may have associated these experiences with their perceptions of actual death, thereby increasing their anxiety. Future studies should thus also probe the valence of the APD experiences and not just their intensity.

Overall, our results, together with the reviewed literature, highlight the transformative nature of psychedelic experiences and their impact on individuals’ perspectives toward death. They contribute to the growing literature emphasizing the critical long-term impact of psychedelic-induced mystical experiences, and call for more research aiming at a more fine-grained understanding of their experiential features.

### 4.2 APDs predict environmental concern

We hypothesized that APD experiences would induce a more selfless mode of psychological functioning as a result of experiencing the self as more flexible (94), thus opening the self to the extended world. Our hypothesis was only partially confirmed. We did not find evidence for reduced self vs. other bias, however, we did find that having experienced APDs predicted higher scores on pro-environmental values and concern. Crucially, ego-dissolution was not predictive of environmental concern, suggesting that among veteran ayahuasca users, APDs are specifically associated with environmental values. The connection between psychedelics and increases in pro-environmental measures such as nature relatedness (21, 95–97), pro-environmental behaviors (98), connection to nature (99), and objective knowledge about climate change (97) has been emerging in the literature. However, the underlying mechanisms remain inadequately explored. To the best of our knowledge, the only studies to date that examine the mechanisms regarding psychedelic-induced increases in pro-environmental attitudes are Lyons & Carhart-Harris (96) and Kettner et al. (21). The latter internet-based prospective study also reported a correlation between heightened nature relatedness and both ego-dissolution as well as the perceived influence of natural surroundings during acute psychedelic states.

One explanation as to why APDs are efficacious in altering environmental attitudes may lie in their efficacy to transform a general conceptual representation of death to a personally-relevant and embodied one. APDs are deeply profound experiences where people have a visceral sense of themselves dying or dead. Such experiences may thus have the potency to break through habitual death denial mechanisms. A recent study (100), adopting a predictive-processing framework, showed that the brain denied death by implementing a powerful and change-resistant top-down prediction that ‘death is related to others’, but not to oneself, thus shielding the self from existential threat. However, the potency and almost ‘real’ nature of APD experiences may be sufficient to penetrate this defensive shield and allow the brain to associate *death* with *self,* thus making the prospect of one’s death more realistic and personally-relevant. This change in encoding might also transform the abstract existential threat of environmental collapse to a personally-relevant visceral threat which must be addressed. In support, recent theoretical papers have linked death defenses and impeding climate action and sustainability (101–103). While this theory requires further validation through longitudinal studies, it provides initial evidence linking APDs to environmental action and concern through the forging of a more realistic, personal and embodied perception of death.

### 4.3 APDs are associated with improved life coping and fulfillment

Several studies provided evidence of enhanced coping abilities among psychedelic users (17, 77, 104, 105), and the modulatory role of 5-HT1A and 5-HT2A receptors in shaping coping styles has been suggested (106). However, the particular experiential aspects that serve as mechanisms of change have received minimal investigation. Here we showed that APD experiences were associated with how stressful situations were coped with. The yAPD group demonstrated higher problem-focused coping scores, compared to the nAPD group, albeit emotion-focused coping did not differ between the two groups. These results are aligned with a previous study demonstrating that hallucinogen usage led to increased problem-focused, but not emotional coping engagement when dealing with the challenges posed by COVID-19 (77). Generally, problem-focused coping involves taking practical steps toward actively addressing the source of stress or problem, while emotion-focused coping focuses on managing and regulating emotions in response to stress without directly addressing the stressor itself (107). While the effectiveness of emotion-focused coping can be influenced by the specific form of strategy employed and various factors and variables, the prevailing consensus in the stress and coping literature is that emotion-focused coping processes are generally maladaptive (107). Problem-focused coping, on the other hand, is generally considered to be an adaptive and constructive approach. Therefore, we can conclude that APDs are associated with enhanced adaptive coping abilities.

Regarding life values, in line with the suggestion that psychedelic-induced personal death experiences lead to transformative changes in life’s values and sense of fulfillment (24), our findings show that the yAPD group reported a significant increase in their sense of life fulfillment, as a result of recognizing and living in accordance with their personal values. These results are likely not resulting from mere ayahuasca intake but rather from the APD experience, as our current findings did not find a correlation between lifetime ayahuasca intake frequency and life values. In support, a recent study (108), utilizing the same measure reported here, also found no difference in life values between controls and ayahuasca users, and no correlation between life values and lifetime ayahuasca intake frequency (but see (76), who did). Thus, it may be the case that the profound changes in life values attributed to ayahuasca (25) may be mediated by APDs. These results complement previous existentially-oriented studies describing increased sense of purpose (109), life meaning (104), and changes in personal values (110) to be associated with psychedelics use. From an existential perspective, the perceived confrontation with mortality acts as a catalyst prompting individuals to reassess their priorities, beliefs, and values, as previously suggested (111). This process of re-evaluation has the potential to facilitate a deeper understanding and fulfillment of personal purpose and ignite a renewed drive and coping abilities to pursue meaningful goals (111).

### 4.4 Study limitations

The current study has several limitations. Firstly, it relies primarily on self-reported measures, which have their inherent limitations. Secondly, the study’s cross-sectional design does not allow the attribution of causality to any of the reported results. Thirdly, the trait measures employed assess only attitudes rather than ‘real-life’ measures of lifestyle and behavior changes. Thus, future studies should employ longitudinal designs and employ also measures of lifestyle and behavioral measures. Ideally, to establish causal effects of APDs while controlling for potential confounds, it would be valuable to conduct interventional clinical studies involving a controlled administration of ayahuasca, meticulously documenting dosage and documenting the occurrence of APDs during the acute state.

Study 1 is also limited by its small sample size and risk for selection bias given its unique sample of veteran ayahuasca users with extensive experience with the brew and ceremonial settings. This limitation was partially addressed by Study 2 which surveyed many more participants, and also did not exclude participants with little experience. Thus Study 2 can be considered as representative of ayahuasca users in Israel. Nevertheless, it is important for future studies to examine APDs in other countries, as well as address other ayahuasca intake settings (e.g., non-ceremonial context). Such an approach would yield a more comprehensive comparison and a deeper exploration of the distinct effects associated with ayahuasca itself, as well as the control of extrapharmacological factors (i.e., set and setting) (112, 113) specifically related to ayahuasca ceremonial use. As previously proposed, extrapharmacological factors may play a significant role in shaping subjective effects of ayahuasca (114) potentially impacting the nature of APDs and their long-term outcomes.

An additional limitation regards the translation of the scales from their original language into Hebrew, with some of the translated tools not undergoing a formal validation process and cultural adaptation. While the practice of reverse translation, as utilized in our study and others, is widely accepted in the literature and cross-cultural research, a formal validation process is recommended.

Finally, we acknowledge a lack of precise definition and rich phenomenological description of the APD experience. As this phenomenon is a profound mystical experience, which may encompass diverse aspects and types of encounters, APDs would benefit from an empirical phenomenological investigation. We anticipate that our forthcoming comprehensive phenomenological study will tease apart personal death experiences from ego dissolution and mystical-type experiences more generally. Future studies might also benefit from incorporating NDE scales, such as the Near-Death Experience Scale (115). This will allow directly examining similarities and differences between APDs and NDEs. This is important as an alternative perspective on our findings could be that some of our observed effects might be linked to mystical experiences in general, which are likewise connected to shifts in perceptions of death (17–20) and highly related to ayahuasca compared to other psychedelics (32). Importantly, this limitation is not relevant in the context of environmental concern, where we showed that ego dissolution did not predict environmental concern.

Despite these limitations, we are confident that the present study makes a significant and innovative contribution to our understanding of APDs and their impact on life, death and the environment. It offers an important addition to the existing literature on psychedelic-induced subjective effects, spotlighting APDs for the very first time. We hope that this study will spark further interest in these profound experiences and further our understanding of the potential they hold for personal and societal transformation.

## Data availability statement

The raw data supporting the conclusions of this article will be made available by the authors, without undue reservation.

## Ethics statement

The studies involving humans were approved by Education Faculty, the University of Haifa, Israel. The studies were conducted in accordance with the local legislation and institutional requirements. The participants provided their written informed consent to participate in this study.

## Author contributions

JD: Conceptualization, Data curation, Formal analysis, Investigation, Visualization, Writing – original draft. JB: Resources and Methodology of study 2, Writing – review & editing. MK: Resources and Methodology of study 2, Writing – review & editing. GO: Resources and Methodology of study 2, Writing – review & editing. NT: Investigation of study 2. TA: Investigation of study 2. YD-Z: Conceptualization, Methodology, Formal analysis, Supervision, Funding acquisition, Writing – original draft. AB-O: Conceptualization, Supervision, Project administration, Funding acquisition, Writing – review & editing.
