# Corrigendum: Ayahuasca-induced personal death experiences: prevalence, characteristics, and impact on attitudes toward death, life, and the environment

**DOI:** 10.3389/fpsyt.2025.1595067

**Published:** 2025-04-01

**Authors:** Jonathan David, José Carlos Bouso, Maja Kohek, Genís Ona, Nir Tadmor, Tal Arnon, Yair Dor-Ziderman, Aviva Berkovich-Ohana

**Affiliations:** ^1^ Edmond J. Safra Brain Research Center, University of Haifa, Haifa, Israel; ^2^ Integrated Brain and Behavior Research Center (IBBRC), University of Haifa, Haifa, Israel; ^3^ Department of Counseling and Human Development, Faculty of Education, University of Haifa, Haifa, Israel; ^4^ International Center for Ethnobotanical Education, Research & Service (ICEERS), Barcelona, Spain; ^5^ Medical Anthropology Research Center (MARC), Department of Anthropology, Philosophy and Social Work, Universitat Rovira i Virgili, Tarragona, Spain; ^6^ Department of Neurosciences and Behavior, University of São Paulo, São Paulo, Brazil; ^7^ Integral Transpersonal Psychology, California Institute of Integral Studies, San Francisco, CA, United States; ^8^ Department of Learning and Instructional Sciences, Faculty of Education, University of Haifa, Haifa, Israel

**Keywords:** ayahuasca, psychedelics, death, self, environmental concern, coping, life fulfillment

In the published article, there was an error regarding the average number of ayahuasca uses in the ayahuasca group in Study 1. A correction has been made to the **Results**, *2.2.1 Participants characteristics*. This sentence previously stated:

“Briefly, on average, our study participants have used ayahuasca (mean = 69.4 ± 98.7), 6.4 times more than psilocybin (mean = 10.7 ± 15.4, U = 1378, p < 0.01, rp = 1), 5.7 times more than mescaline (mean = 12 ± 14.9, U = 351, p < 0.01, rp = 1), and 7.07 times more than LSD (mean = 9.9 ± 16.6, U = 976, p < 0.01, rp = 1).”

The corrected sentence appears below:

“Briefly, on average, our study participants have used ayahuasca (55.7 ± 82.1), 5.2 times more than psilocybin (mean = 10.7 ± 15.4, U = 1378, p < 0.01, rp = 1), 4.6 times more than mescaline (mean = 12 ± 14.9, U = 351, p < 0.01, rp = 1), and 5.6 times more than LSD (mean = 9.9 ± 16.6, U = 976, p < 0.01, rp = 1).”

In the published article, there was an error in **Figure 2** and **Figure 3** as published. The authors inadvertently used SD (Standard Deviation) in the bar plots in the figures instead of SEM (Standard Error of the Mean). In the original captions we reported SEM. The corrected **Figure 2** and **Figure 3** and their captions appear below.

**Figure 2:**
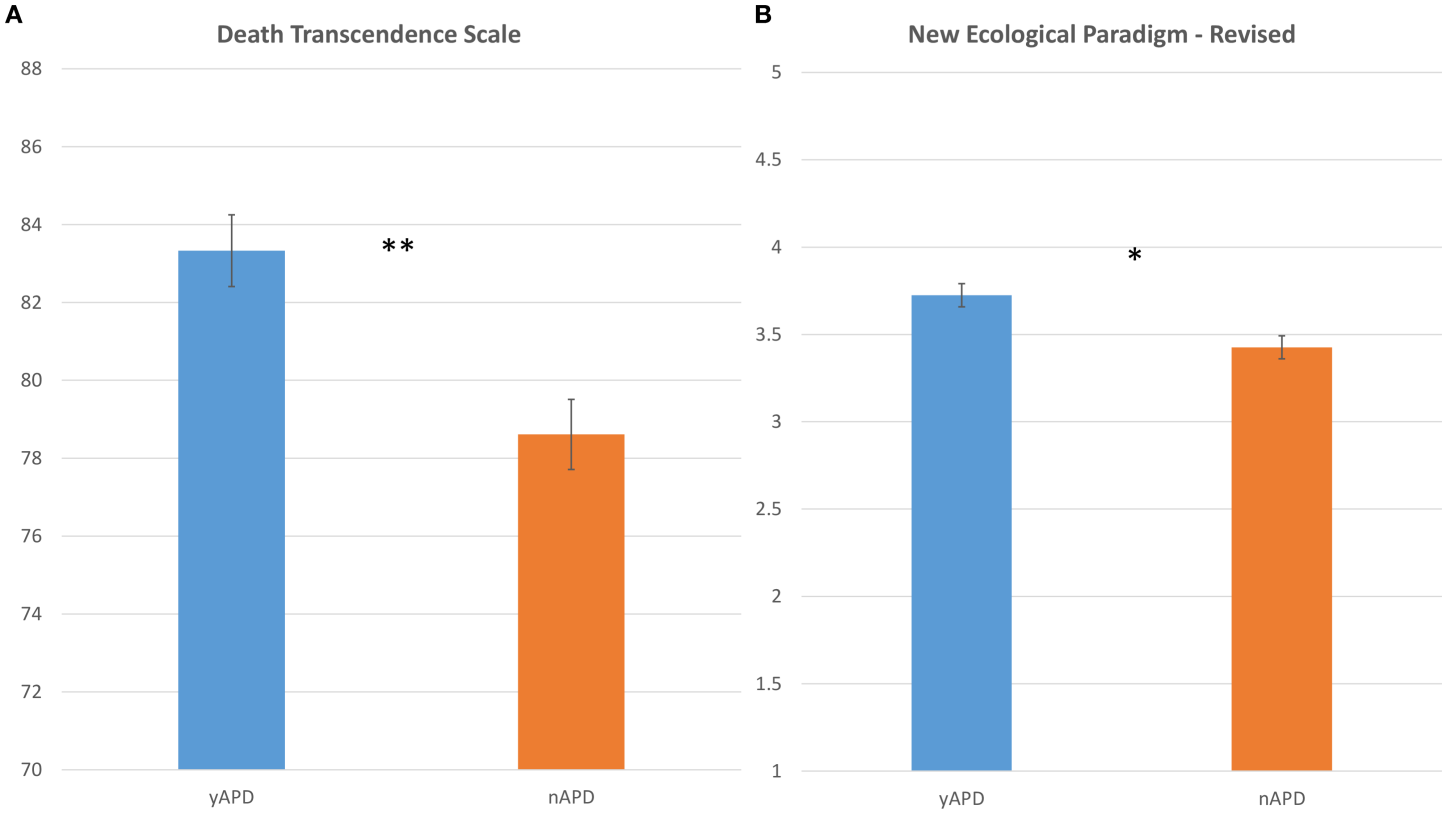
Death transcendence attitudes and environmental concern as a function of experiencing APDs. Bar plots comparing the distribution of **(A)** DTS scores (y-axis), and **(B)** NEP-R scores (y-axis), as a function of the yAPD group (in blue) and nAPD group (in orange). Error bars represent the standard error of the mean. DTS, Death Transcendence Scale, NEP-R, New Environmental Paradigm Revised. Statistics: p-values <=0.01 are denoted by **, and p-values <=0.05 are denoted by *.

**Figure 3:**
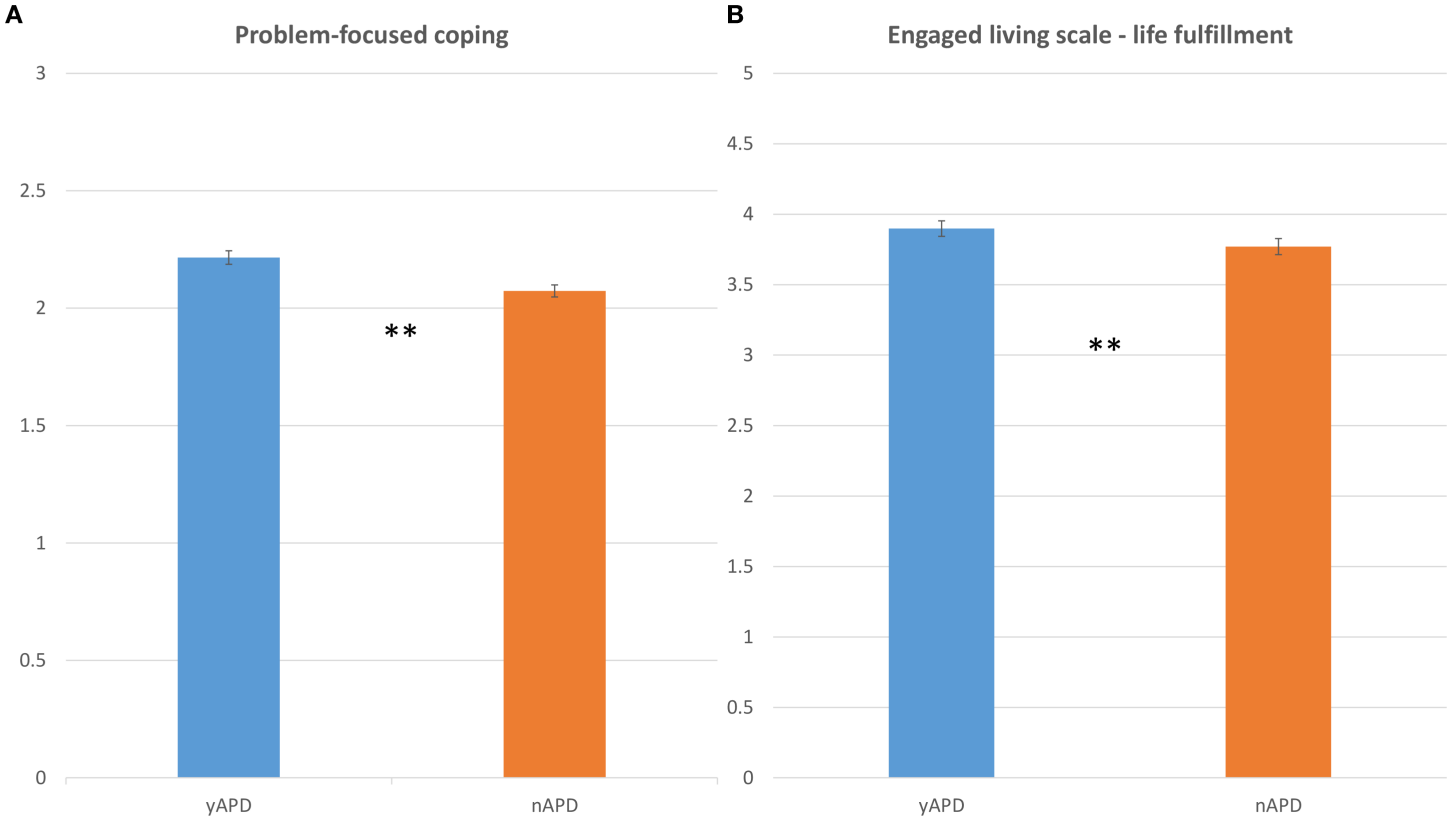
Life engagement and coping strategies as a function of APD. Bar plots comparing the distribution of **(A)** COPE-p scores (y-axis), and **(B)** ELS-f scores (y-axis), as a function of the yAPD group (in blue) and nAPD group (in orange). Error bars represent the standard error of the mean. Abbreviations: COPE-p, Problem-focused coping; ELS-f, Engaged Living Scale-life fulfillment. Statistics: p-values <=0.01 are denoted by **, and p-values <=0.05 are denoted by *.

The authors apologize for these errors and state that this does not change the scientific conclusions of the article in any way.

