# Supplementary material for: Ayahuasca-induced personal death experiences: prevalence, characteristics, and impact on attitudes toward death, life, and the environment
Source: Front Psychiatry. 2023 Dec 19;14:1287961. doi: 10.3389/fpsyt.2023.1287961 (PMC10758466; doi:10.3389/fpsyt.2023.1287961)
Supplement: Supplementary file 1 [file Table_1.docx]

**Supplementary Material**

**Supplementary Table 1: The table presents the lifetime use of psychedelic drugs, including Ayahuasca, Mescaline, LSD, and Psilocybin in Study 1. A comparison is provided between yAPD and nAPD groups within each drug category. Mann-Whitney U test was performed. Results reveal no significant differences between yAPD and nAPD for any psychedelic drug.**

| Drug | yADP | nADP | Total | Statistics (all n.s.) |
| --- | --- | --- | --- | --- |
| ayahuasca | N=36 | N=18 | N=54 | U=255 |
|  | 69.4±97.8 | 28.5±13.7 | 55.8 ±82.1 |  |
| LSD | N=32 | N=11 | N=43 | U=166 |
|  | 11.3±18.9 | 6.2±5.3 | 9.9±16.6 |  |
| Mescaline | N=20 | N=6 | N=26 | U=59 |
|  | 26 | 8.9 | 12±14.9 |  |
| Psilocybin | N=36 | N=16 | N=52 | U=225 |
|  | 12.5±18 | 6.5±5.2 | 10.7±15.4 |  |

**Supplementary Table 2**: **the table shows a comparison of lifetime use of Psilocybin, Mescaline, and LSD between the yADP and nADP groups in study 2. The values in the table represent the frequency of use reported by participants in each group. The statistical test used for comparison was Mann-Whitney U test. Bold markings indicate statistically significant findings.**

|  |  | yADP | nADP | Total | Statistics |
| --- | --- | --- | --- | --- | --- |
|  |  | N=150 | N=155 | N=305 |  |
|  | Never | 39 (12.8%) | 47 (15.4%) | 86 (28.2%) |  |
|  | 1-2 | 23 (7.5%) | 22 (7.2%) | 45 (14.8%) |  |
|  | 3-5 | 16 (5.2%) | 22 (7.2%) | 38 (12.5%) |  |
| LSD | 6-9 | 15 (4.9%) | 14 (4.6%) | 29 (9.5%) | U=10904  p=n.s |
|  | 10-19 | 24 (7.9%) | 18 (5.9%) | 42 (13.8%) |  |
|  | 20-29 | 13 (4.3%) | 14 (4.6%) | 27 (8.3%) |  |
|  | 30+ | 20 (6.6%) | 18 (5.9%) | 38 (12.5%) |  |
|  |  |  |  |  |  |
|  | Never | 11 (3.6%) | 25 (8.2%) | 36 (11.8%) |  |
|  | 1-2 | 13 (4.3%) | 22 (7.2%) | 35 (11.5%) |  |
|  | 3-5 | 18 (5.9%) | 12 (3.9%) | 30 (9.8%) |  |
| Psilocybin | 6-9 | 23 (7.5%) | 26 (8.5%) | 49 (16.1%) | **U=9278**  **P<0.01** |
|  | 10-19 | 32 (10.5%) | 39 (12.8%) | 71 (23.3%) |  |
|  | 20-29 | 21 (6.9%) | 13 (4.3%) | 34 (11.1%) |  |
|  | 30+ | 32(10.5%) | 18 (5.9%) | 50 (16.4%) |  |
|  |  |  |  |  |  |
|  | Never | 32 (10.5%) | 60 (19.7%) | 92 (30.2%) |  |
|  | 1-2 | 31 (10.2%) | 27 (8.7%) | 58 (19%) |  |
|  | 3-5 | 27 (8.9%) | 19 (6.2%) | 46 (15.1%) | **U=9518**  **P<0.01** |
| Mescaline | 6-9 | 15 (4.9%) | 12 (3.9%) | 27 (8.9%) |  |
|  | 10-19 | 17 (5.6%) | 17 (5.6%) | 34 (11.1%) |  |
|  | 20-29 | 10 (3.3%) | 7 (2.3%) | 17 (5.6%) |  |
|  | 30+ | 18 (5.9%) | 13 (4.3%) | 31 (10.2%) |  |
